# Comparison of stereopsis thresholds measured with conventional methods and a new eye tracking method

**DOI:** 10.1371/journal.pone.0293735

**Published:** 2023-11-02

**Authors:** Lu Liu, Bo Yu, Lingxian Xu, Shiyi Wang, Lingzhi Zhao, Huang Wu

**Affiliations:** Department of Optometry, The Second Hospital of Jilin University, Changchun, China; The Ohio State University, UNITED STATES

## Abstract

**Purpose:**

Stereopsis is the ability to perceive depth using the slightly different views from two eyes. This study aims to conduct innovative stereopsis tests using the objective data outputted by eye tracking technology.

**Methods:**

A laptop and an eye tracker were used to establish the test system. Anaglyphic glasses were employed to execute the stereopsis assessment. The test symbol employed was devised to emulate the quantitative measurement component of the Random Dot 3 Stereo Acuity Test. Sub-pixel technology was used to increase the disparity accuracy of test pages. The tested disparities were: 160″, 100″, 63″, 50″, 40″, 32″, 25″, 20″, 16″, and 12.5″. The test was conducted at a distance of 0.65m. Conventional and eye tracking stereopsis assessments were conducted on 120 subjects. Wilcoxon signed-rank test was used to test the difference, while the Bland-Altman method was used to test the consistency between the two methods.

**Results:**

The Wilcoxon signed-rank test showed no significant difference between conventional and eye tracking thresholds of stereopsis (*Z* = −1.497, *P* = 0.134). There was a high level of agreement between the two methods using Bland- Altman statistical analysis (The 95 per cent limits of agreement were −0.40 to 0.47 log arcsec).

**Conclusions:**

Stereoacuity can be evaluated utilizing an innovative stereopsis measurement system grounded in eye tracking technology.

## Introduction

Stereopsis, also referred to as stereoscopic vision or depth perception, is the capacity of the brain to discern three-dimensional (3D) depth from the marginally distinct two-dimensional (2D) images projected onto the retinas of each eye. This variation, known as binocular disparity, allows humans and certain other animals to perceive the relative distance between objects and evaluate their depth within the environment [1]. The function of stereopsis is assessed by the threshold of stereopsis, that is, stereoacuity.

Numerous clinical assessments have been devised to appraise stereopsis or depth perception. These evaluations are often designed to be straightforward, rapid, and effortless to administer, facilitating healthcare professionals in assessing a patient’s binocular vision and diagnosing potential visual disorders. Instruments employed for this purpose include the Titmus Fly Stereotest [2], TNO Stereotest [3, 4], Randot Stereotest [5–7], Frisby Stereotest [8], and Lang Stereotest [9]. These tests are routinely utilized in clinical settings to assess stereopsis, identify visual disorders, and track treatment progress. They offer invaluable insights into an individual’s depth perception, which is crucial for various daily tasks and activities.

However, all the tests mentioned above in clinical practice require direct communication between the examiner and the assessed individual. Subjects cannot undergo a self-administered test during the examination without medical personnel intervention.

Several methods exist for objectively evaluating stereopsis, such as electrophysiological measurements and functional magnetic resonance imaging (fMRI) [10–12]. Techniques including electroencephalography (EEG) [13, 14], magnetoencephalography (MEG) [15, 16], and event-related potentials (ERPs) [17] may be utilized to investigate the neural responses correlated with stereoscopic processing. By scrutinizing specific patterns of neural activity, researchers can objectively appraise an individual’s depth perception capabilities. FMRI is a non-invasive approach employed for studying brain activity associated with stereoscopic vision [18]. By analyzing the activation of particular brain regions during stereoscopic stimuli presentation, researchers can objectively assess an individual’s depth perception. These objective methods offer means to evaluate stereopsis without relying on the subject’s responses or feedback, yielding a more precise and reliable assessment of depth perception capabilities. However, these methods can be complex and challenging to implement in clinical settings.

Eye tracking technology has experienced significant advancements since its inception in the early 20th century. Eye trackers are non-invasive devices that capture and analyze eye movements, providing insight into the visual and oculomotor systems. In recent years, eye tracking technology has been employed in numerous ophthalmological applications, from monitoring and diagnosing ocular disorders to enhancing surgical outcomes and devising new diagnostic tools [19, 20].

In diagnostics, eye trackers have emerged as helpful instruments in ophthalmology, serving various applications [21]. They can assess ocular motility and detect disorders such as strabismus and nystagmus [22–25], evaluate binocular vision by examining eye coordination [26], and assist in visual field testing to monitor glaucoma progression and other visual impairments [27, 28]. Furthermore, eye trackers can analyze reading difficulties or dyslexia by observing eye movements, fixations, and saccades during reading tasks [29, 30]. Overall, eye trackers enhance diagnostic accuracy and provide essential data to optimize patient care and treatment.

In the research domain, eye trackers significantly impact ophthalmology by offering valuable insights into eye diseases and disorders [31, 32]. They aid researchers in investigating the pathophysiology of various eye conditions (e.g., amblyopia, strabismus) and their effects on vision by furnishing detailed data on eye movement patterns and gaze behavior [33]. Moreover, eye trackers can be employed to evaluate the efficacy of treatments and interventions by comparing changes in eye movement patterns pre- and post-therapy or procedures [34]. In sum, eye trackers contribute to ophthalmology research, fostering the development of innovative diagnostic techniques and novel treatments for an extensive range of eye conditions.

Within the sphere of surgical assistance and training, eye trackers have proven useful in ophthalmology [35]. They can be integrated with robotic surgical systems to improve precision by utilizing the surgeon’s gaze data to control robotic instruments [36]. Furthermore, eye trackers can enhance surgical navigation by providing real-time feedback on the location of surgical instruments relative to ocular structures. In training and education, eye trackers are employed to evaluate and refine the performance of ophthalmology residents and medical students during surgical simulations or live surgeries, assisting them in developing superior situational awareness and procedural skills [37–39]. Applications of eye trackers contribute to improved surgical outcomes and patient care in ophthalmology.

In vision rehabilitation, eye trackers have demonstrated their utility in augmenting the efficacy of treatments and assistive technologies for patients with visual impairments [40]. By providing biofeedback, eye trackers can be utilized in vision therapy to offer real-time feedback on eye movements, enabling patients to learn how to enhance their eye coordination and control [41]. Additionally, eye trackers can be integrated with assistive devices such as screen readers, allowing individuals with visual impairments or motor disabilities to browse digital content more effectively [29]. These applications of eye trackers contribute to improved visual function and quality of life for patients undergoing vision rehabilitation.

Despite considerable progress in the field of ophthalmology, there is a paucity of research concentrating on eye movement characteristics during stereopsis assessments. Stereopsis measurement using eye tracking technology offers its greatest advantages in scenarios where traditional test methods fall short, proving ineffective, unsuitable, or unfeasible. In upcoming applications, it could serve as a valuable technique in situations with communication barriers between the examiner and the individual, such as when evaluating infants, preverbal children, those with speech impediments, or individuals suspected of feigning symptoms. In this study, we endeavor to employ an eye tracker to evaluate the measurement of stereopsis in a non-verbal manner innovatively. This method is compared with the conventional test, which requires supervision by medical personnel and a verbal response from the patient. The goal is to offer novel insights in the realm of stereopsis measurement.

## Materials and methods

### Equipment

**Fig 1:**
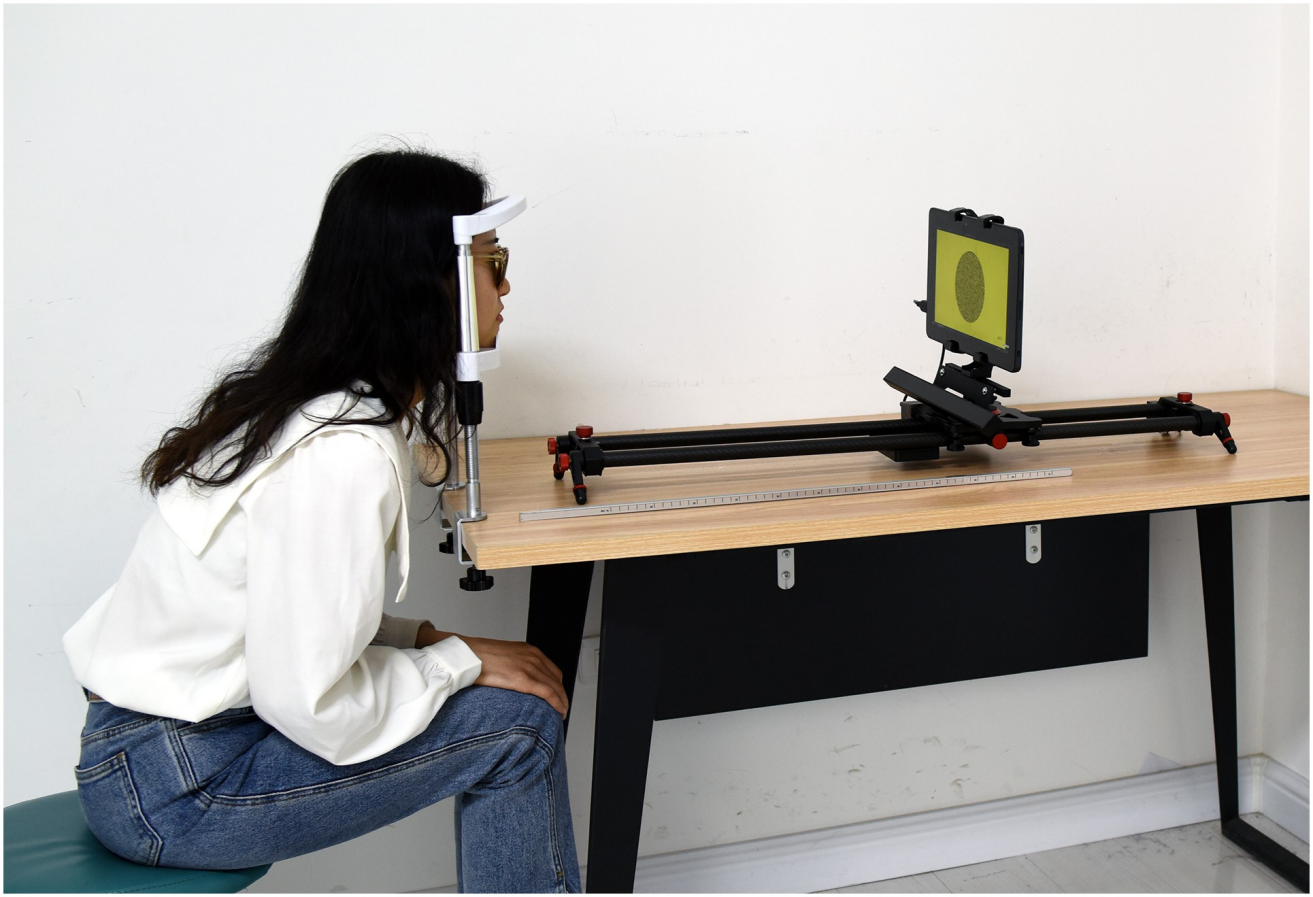
Photograph of the stereoacuity test system combined with the eye tracker.

An experimental configuration was implemented, employing a Dell Latitude 5175 laptop (produced by Dell [China] Co., Ltd.) outfitted with a 10.8-inch display screen boasting a resolution of 1920 × 1080 pixels. The display featured a dot pitch of 0.12mm, thereby generating a disparity of 40 seconds of arc (40") with a single-pixel disparity at a distance of 0.65 meters. Ocular movements were meticulously documented using the Eyeso Ec80 eye tracker (fabricated by Braincraft Technology Co., Ltd.), and the following data were systematically analyzed via Eyeso Studio V5.0 software. The Eye tracker is equipped with a sampling rate of 90 Hz and a spatial resolution of 0.5°. Anaglyphic glasses were employed to execute the stereopsis assessment, explicitly utilizing the TNO stereotest. The eye tracker accurately detected ocular movements, even while participants wore spectacles (Fig 1).

### Test symbol

**Fig 2:**
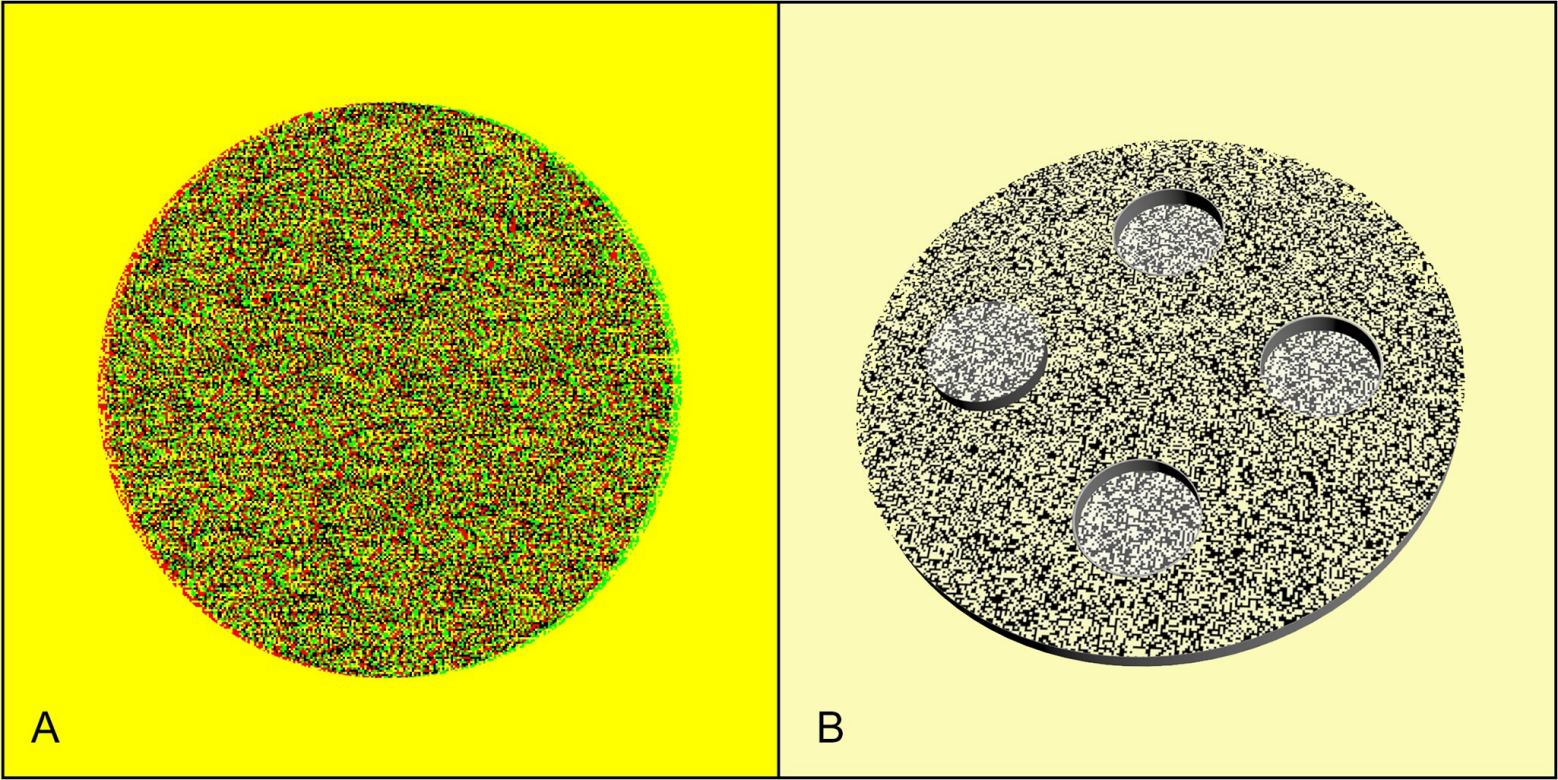
Illustration of a test page legend. A represents the single-eye perspective of the test page. B simulates the stereo image perceived when the test pages viewed by the left and right eyes were fused correctly. The left prominent ring was designated as the target symbol.

In this study, the test symbol employed was devised to emulate the quantitative measurement component of the Random Dot 3 Stereo Acuity Test (Vision Assessment Corporation, Illinois, USA). The symbol comprised a sizable disc with a diameter spanning 800 pixels, accompanied by four smaller circles, each measuring 160 pixels in diameter, symmetrically positioned in the cardinal directions of up, down, left, and right. Among these four circles, one was designated as the target symbol, exhibiting crossed disparity, while the remaining three served as control targets characterized by uncrossed disparity. The purpose of this test is to identify the circle that protrudes out of the plane (Fig 2).

Sub-pixel technology was utilized in the study. All test symbols were generated through a program written in C# combined with the development kit of OpenCV 3.0. The bilinear interpolation algorithm was used to implement sub-pixel shifts of the test symbols. Disparities were established at the following values: 160" (2.2 log arcsec), 100" (2 log arcsec), 63" (1.8 log arcsec), 50" (1.7 log arcsec), 40" (1.6 log arcsec), 32" (1.5 log arcsec), 25" (1.4 log arcsec), 20" (1.3 log arcsec), 16" (1.2 log arcsec), and 12.5" (1.1 log arcsec). Correspondingly, these values represented disparities of 4-pixel, 2.5-pixel, 1.6-pixel, 1.3-pixel, 1-pixel, 0.8-pixel, 0.6-pixel, 0.5-pixel, 0.4-pixel, and 0.3-pixel.

### Establishment of test indices

#### Stereopsis recognition reaction time

*Participants*. A total of 30 participants (20 female and 10 male, aged 21 to 50 years), were recruited for a pre-experiment to obtain the stereopsis recognition reaction time. Each participant exhibited a visual acuity of no less than 0 logMAR in each eye, along with a stereoacuity of no less than 32″, as assessed by the Fly Stereo Acuity Test (Vision Assessment Corporation, Illinois, USA). The research conformed to the ethical principles delineated in the Declaration of Helsinki and received approval from the Ethics Committee of the Second Hospital of Jilin University (Approval No. 2022–119).

*Test procedure*. Participants were instructed to position their heads securely in the provided bracket, ensuring a consistent viewing distance of 0.65 meters from the screen. Test pages were displayed on the screen, and participants were tasked with identifying the position of the target circle. Each choice was evaluated against the correct answer to ascertain whether the participant succeeded or faltered on the test page. The interval between the test page’s appearance and the successful detection of the target was recorded as the stereopsis recognition reaction time, utilizing a stopwatch operated by the participants. The test page’s disparity began at 160″ and was incrementally reduced until the participant could no longer accurately discern the target’s location. The final correct disparity served as the participant’s threshold, and the associated stereopsis recognition reaction time was duly recorded.

#### Defining the scope of the area of interest (AOI)

**Fig 3:**
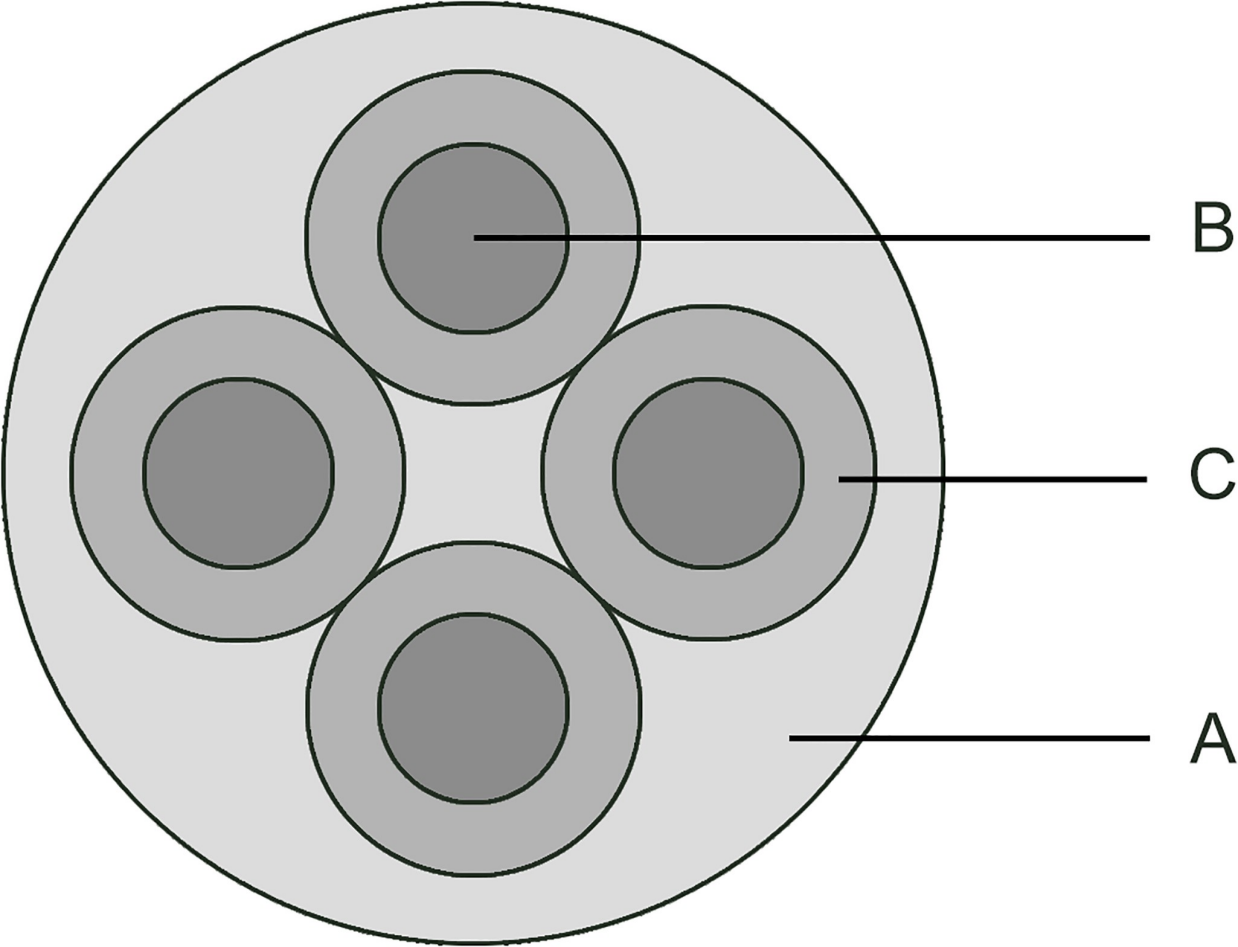
Illustration of the area of interest (AOI). There are three circles: large circle A with a diameter of 800 pixels, small circle B with a diameter of 160 pixels, and medium-sized circle C with a diameter of 283 pixels. The large circle A represents the whole range of the random-dot test page. The small circle B depicts the area where the stereoscopic target is positioned. Each small circle’s area makes up 4% of the large circle A. The middle solid circle C is the set AOI range, and the area of each middle circle accounts for 12.5% of the large circle A.

Considering the presence of four smaller circles within the large circle, four distinct AOIs were established, corresponding to their respective positions. Each AOI encompassed a circular region with a diameter of 283 pixels, situated at the up, down, left, and right positions (refer to area C in Fig 3) within the large circle. During the analysis, the AOI where the target symbol located in is regarded as the target AOI.

#### Utilizing eye movement indices to determine stereopsis

This test aims to enable participants to identify and continuously focus on the target. When examining the test page, subjects typically scan the page in search of the target. Upon discovering the target, they cease scanning and concentrate on it. If the tested disparity falls below the subject’s threshold, the subject generally continuously scans the test surface without fixating on any specific point. If the tested disparity considerably exceeds the subject’s threshold, scanning time is reduced, and the subject’s gaze quickly locks onto the target. As the threshold is approached, uncertain scanning motions usually precede target identification, after which the gaze can be sustained.

In this test, the fixation rate on the AOI was employed to determine the subject’s confirmation of the target. The Fixation rate on AOI refers to the frequency of the fixation duration on a specific AOI during an eye tracking session.

In this experiment, the AOI’s area constitutes 12.5% of the large circle’s area. Consequently, the fixation rate on AOI before the detection of stereoscopic targets is approximately 12.5%. Following the detection of stereoscopic targets, the fixation rate on AOI rises to approximately 100%. Theoretically, the fixation rate on AOI can be expressed as (target detection time × 12.5% + gaze time) / (target detection time + gaze time). Under random scanning conditions, the fixation rate on AOI remains at 12.5%. When the fixation rate on AOI reaches 50%, it can be reasonably concluded that the subject has discerned the stereopsis. After establishing the target detection time, it is feasible to calculate the total test time at which the fixation rate on AOI achieves 50%.

#### Comparison between conventional and eye tracking thresholds of stereopsis

*Participants*. This study was conducted in the Eye Hospital of the Second Hospital of Jilin University, China, from September 2022 to April 2023. 120 participants, aged between 21 and 50, were enrolled in the study. Each participant exhibited a stereoacuity of no less than 160″, as assessed by the Random Dot 3 Stereo Acuity Test.

Before participation in the study, a written, informed consent was obtained from all participants. The research adhered to the ethical principles delineated in the Declaration of Helsinki and received approval from the Ethics Committee of the Second Hospital of Jilin University (Approval No. 2022–119).

*Conventional assessment of stereopsis thresholds*. The disparity of the test page commenced at 160″ and was incrementally reduced until participants could no longer accurately identify the targets. The final correct disparity was recorded as the participant’s threshold.

*Eye tracking assessment of stereopsis thresholds*. Test pages were prepared in the following order: 160″, 100″, 63″, 50″, 40″, 32″, 25″, 20″, 16″, and 12.5″. The duration of a test page was determined experimentally, as described above. A one-second blank image was displayed between each test page, signifying the end of the current disparity test and the imminent presentation of the subsequent disparity. Participants were instructed to locate the protruding circles on the test page and maintain their gaze upon them. For a single disparity test page, the result was judged as pass when the fixation rate on AOI was higher than 50%; otherwise, recorded as a failure.

### Data analysis

PASW Statistics 26.0 (SPSS Inc., Chicago, IL, USA) was used to process the data. The Shapiro-Wilks or Kolmogorov Smirnova test was used to investigate the data distribution. A parametric test, more precisely, the paired t-test, was used to identify group differences in the data with a normal distribution. However, the nonparametric Wilcoxon signed-rank test was applied, for data that did not follow the normal distribution. To assess the agreement, the Bland-Altman method was implemented (version 20.0, MedCalc Software bvba, Ostend, Belgium). For statistical significance, a significance level of *P* <0.05 was used. Stereopsis values were transformed to log arcsec values for analysis.

## Result

### Stereopsis recognition reaction time

**Fig 4:**
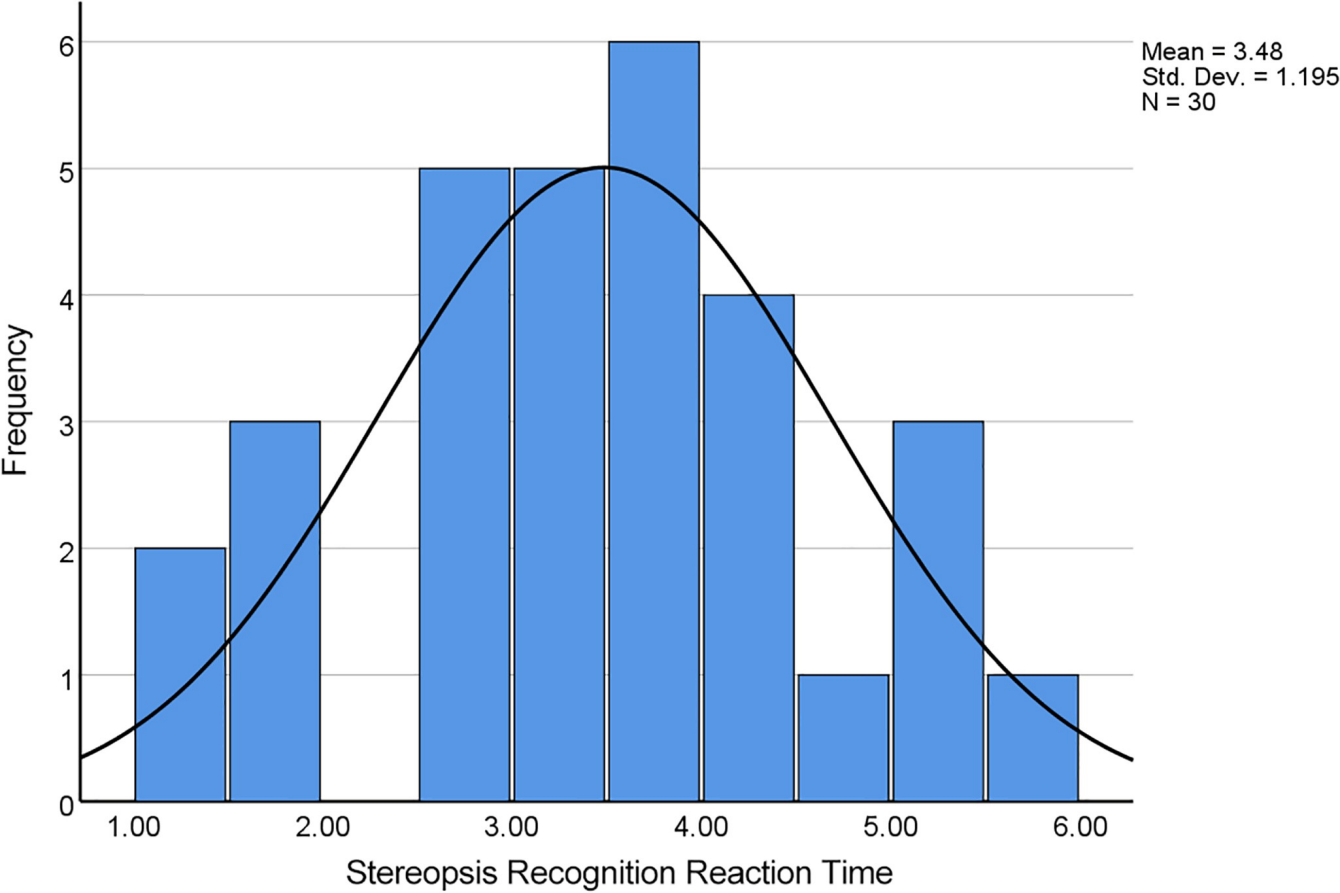
Histogram of the stereopsis recognition reaction time data.

The pre-experiment data distribution conforms to a normal distribution (Shapiro-Wilk test, *P* = 0.769) (Fig 4). Stereopsis recognition reaction time is 3.48±1.19 seconds. The maximum stereopsis recognition reaction time was 5.82 seconds. This value was used to calculate the total test time.

In the eye tracking (non-verbal) assessment of the stereopsis thresholds, the total test time of a single disparity test page is equal to target detection time plus gaze time. The maximum stereopsis recognition reaction time was utilized as the target detection time, and the effective fixation rate on AOI was set as 50%. Therefore, the total test time could be calculated using the theoretical formula mentioned above: total test time = 1.75×target detection time, which is 10.19 seconds. In practice, 10 seconds was adopted as the total test time of a single disparity test page.

### Comparison between conventional and eye tracking thresholds of stereopsis

**Fig 5:**
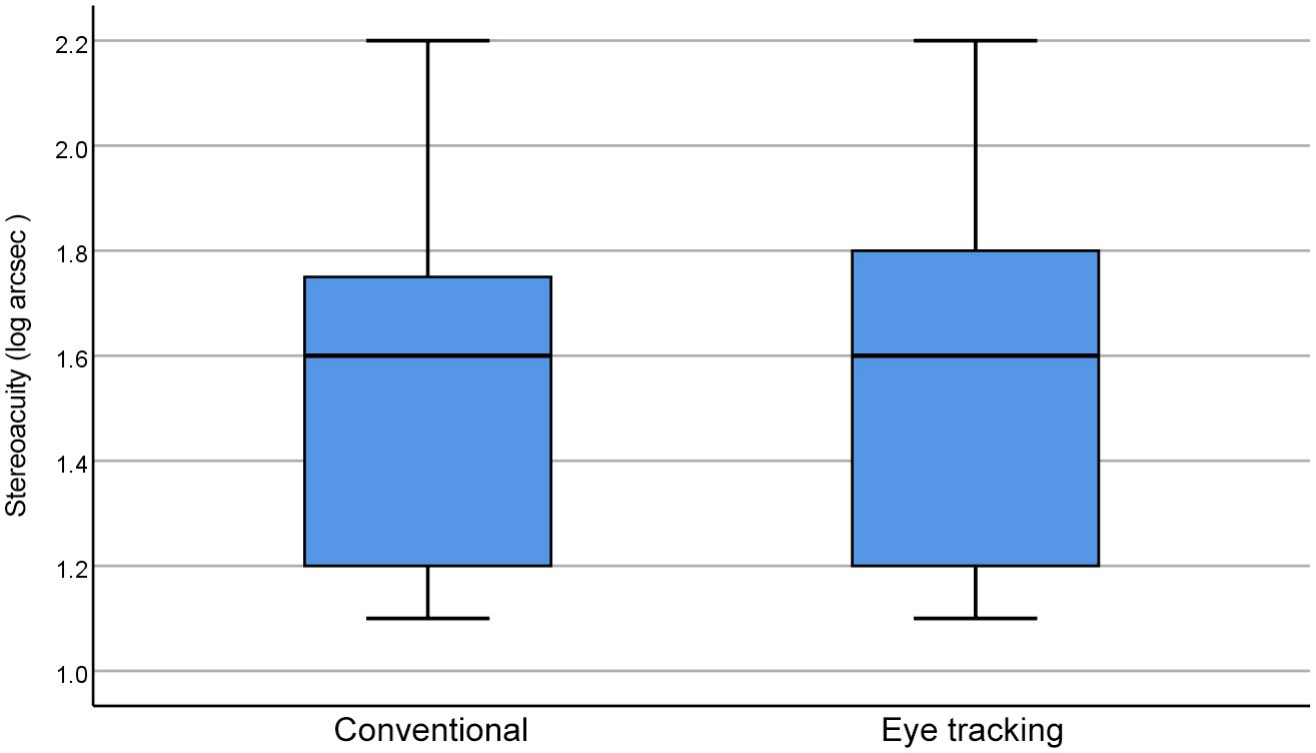
Boxplot of conventional and eye tracking thresholds of stereopsis. The line perpendicular to the whisker below the box represents the minimum value; the lines of the box represent the interquartile range (the lower edge represents the first quartile; the upper edge represents the third quartile); the line in the box represents the median value; the line perpendicular to the whisker above the box represents the maximum value.

**Fig 6:**
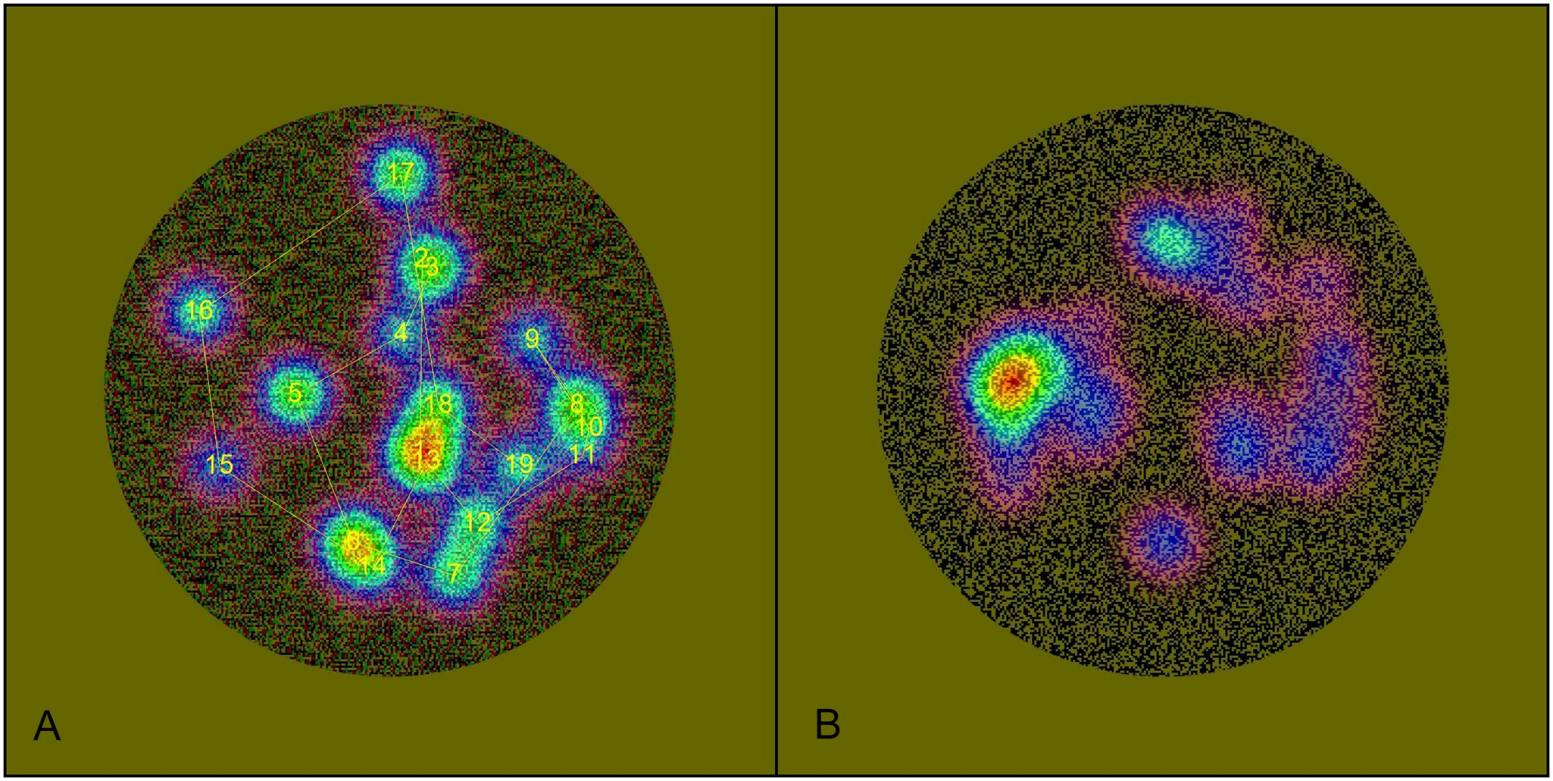
Examples of heat maps in innovative stereopsis thresholds test using an eye tracker. A is the heat map when the disparity of the test page is below the participant’s stereopsis threshold. The lines and numbers in it represent the trajectory and order of the participant ’s fixation points. The participant cannot correctly identify the target; that is, the fixation rate on target AOI does not reach 50%. B is the heat map when the disparity of the test page exceeds the participant’s stereopsis threshold. The participant can correctly identify the target, that is, the fixation rate on target AOI exceeding 50%.

The data distribution didn’t conform to a normal distribution (Kolmogorov Smirnova test, *P*<0.001 in both conventional and innovative eye tracking thresholds of stereopsis). The Wilcoxon signed-rank test showed no significant difference between conventional and eye tracking thresholds of stereopsis (*Z* = −1.497, *P* = 0.134). The boxplot of the conventional (verbal) and eye tracking (non-verbal) thresholds of stereopsis is shown in Fig 5. The median and interquartile range (IQR) of the verbal data was 1.6(0.57) seconds. The median and IQR of the non-verbal data was 1.6(0.6) seconds. The heat maps of the non-verbal thresholds of stereopsis assessment are shown in Fig 6.

**Fig 7:**
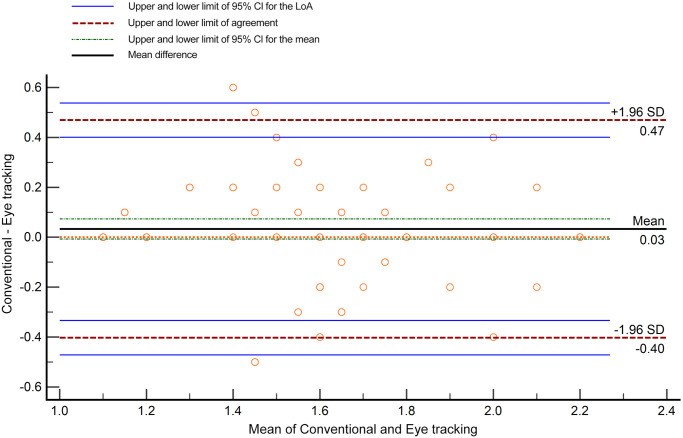
Bland-Altman plots for comparison between the conventional and eye tracking thresholds of stereopsis. The mean difference between methods was 0.03 log arcsec, and the 95% confidence interval (CI) of the mean was −0.007 to 0.074 log arcsec. The 95% limit of agreement (LoA) was −0.40 to 0.47 log arcsec; the 95% CI limit of the lower LoA was −0.472 to −0.334 log arcsec; the 95% CI limit of the higher LoA was 0.400 to 0.538 log arcsec.

The mean of verbal thresholds of stereopsis was 1.565 ± 0.302 log arcsec, compared with 1.532 ± 0.331 log arcsec of non-verbal thresholds of stereopsis. The 95 per cent limits of agreement (LoA) were -0.403 to 0.469 log arcsec (the range equivalent to 7.4 ″). The maximum allowed difference between methods (the interval between the lower 95 per cent confidence interval [CI] limit of the lower LoA and the higher 95 per cent CI limit of the higher LoA 95 per cent CI) was −0.472 to 0.538 log arcsec (the range equivalent to 10.2 ″) (Fig 7).

The LoA of the verbal and non-verbal thresholds of stereopsis (± 0.436 log arcsec, equivalent to ± 2.7 ″) and the maximum allowed difference between methods (± 0.505 log arcsec, equivalent to ± 2.7 ″) in our research are much smaller than the measurement precision of clinical stereopsis tests. Therefore, the two measurements showed a high level of agreement.

## Discussion

Currently, the clinical assessment of stereopsis relies on entirely subjective methods, in which the examiner communicates the test purpose to the subject, and subsequently allows the subject to exam the test pages and actively provide feedback. For instance, in the Frisby near stereotest, the subject is required to inform the examiner which of the four squares contains a protruding or concave circular pattern; in the Titmus stereotest, the subject must identify which of the four circles appears most prominent; in the TNO stereostest, the subject needs to indicate the direction in which "Pac-Man’s mouth" is facing. Although there exist some objective methods for evaluating stereopsis, such as functional magnetic resonance imaging (fMRI), electroencephalography (EEG), and magnetoencephalography (MEG), their application is restricted due to the complexity of these procedures.

The eye tracker serves as an instrument for the objective evaluation of a subject’s eye movement characteristics, offering a simple and non-invasive assessment process that is readily accepted by subjects. By analyzing the eye movement characteristics of the subject, it can be determined whether they have successfully identified the correct test target. Throughout the examination process, the subjects complete the test in accordance with the requirements without providing any verbal information to the examiners. At the completion of the examination, a result judgment is made based on the subject’s response to the test pages. The fixation rate on AOI represents the frequency at which participants fixate on a specific AOI during eye tracking. When elucidating the experiment’s purpose to the subjects, they were instructed to locate and gaze at the prominent target symbols. If the subject’s gaze is concentrated within the target AOI, it can be inferred that the subject has performed according to our instruction.

The following experimental indices were designed: the diameter of the large circle (refer to area A in Fig 3) was 800 pixels, while the diameter of the target circle (refer to area B in Fig 3) was 160 pixels. The ratio of the area between a target circle and the large circle is 4%. Considering the characteristics of the subject’s fixation, it is difficult for them to keep their gaze entirely within the small target circle range even if they perceive stereopsis (Fig 6B). The presence of eye movements, such as micro saccades, may cause the fixation to extend outside the small circle range to a certain extent, even though the subject identifies the correct position of the target symbol. Given the dispersed arrangement of the four small circle test symbols, expanding the scope of the AOI would increase the sensitivity of the test. The AOI is defined as a circle with a diameter of 283 pixels, sharing the same center of the small circle. This definition establishes four AOIs corresponding to the four target circles. Each AOI occupies 12.5% of the large circular area. The fixation point within the corresponding AOI is regarded as effective fixation to calculate the fixation ratio. The ratio should theoretically be 12.5% if the subject failed to identify the test target. That is, if the subject lacks subjective intentional control, the probability of random fixation within the correct AOI range should be 12.5%. If the fixation rate on AOI is adjusted to 25%, the probability of reaching this value at random is 1.56%. If the rate is adjusted to 50%, the probability of reaching this value at random is 0.02%. In other words, when we set the fixation rate on AOI to 50%, there is practically no chance that individuals will complete the task in a random state. If the fixation rate of the AOI is required to exceed 50%, the subject must intentionally keep his fixation point within the AOI area. According to the indices established, the results obtained through the subject’s fixation rate on AOI are theoretically equivalent to the subject subjectively indicating the position of the target circle in the random-dot stereogram.

Correlating verbal assessment with non- verbal assessment: (1) In the non- verbal assessment, if the subject’s fixation rate on AOI exceeds 50% within the specified time, and the subject’s gaze target corresponds with the correct answer, it is equivalent to a successful assessment, wherein the subject accurately identifies the location of the target circle. (2) In the non- verbal assessment, if the subject’s fixation rate on AOI does not exceed 50% within the specified time, it is equivalent to the subject being unable to pinpoint the position of the target circle in the assessment. (3) In the non- verbal assessment, if the subject’s fixation rate on AOI exceeds 50% within the specified time but the subject focuses on an incorrect target, it is equivalent to the subject indicating an incorrect target circle location in the assessment.

According to our results, there is no statistically significant difference between the outcomes of the non- verbal assessment utilizing the eye tracker and the outcomes of the verbal assessment, with a high degree of consistency between the two methods. This finding indicates that for the participants in this study, the outcomes of both tests are essentially equivalent. It demonstrates the feasibility of employing eye tracker technology for conducting non- verbal assessments of stereoscopic vision.

The selection of indices is relatively conservative to validate the effectiveness of the method in this experiment. For instance, the test time for a single disparity page was not based on the average time from theoretical calculations, but rather on the longest reaction time observed within the participants. Utilizing the average time (3.5 seconds) to calculate the test time for a single disparity page would result in a value of 6.1 seconds, which is 3.9 seconds shorter than the time calculated according to the longest reaction time in this study. We adopted a strict threshold level of 50% for the fixation rate on the AOI. It implies that for 50% of the test duration, the fixation is positioned inside the AOI, which accounts for 12.5% of the entire large circle’s area. The error probability for this option is only 0.02%. If the threshold level of fixation rate on the AOI was set at 25%, the error probability would be 1.56%. With a 25% fixation rate on the AOI as the threshold level and the longest reaction time of 5.8 seconds, the calculated single test duration is 6.8 seconds.

It is evident that reducing target detection time and the fixation rate on the AOI can decrease the total test time required for the experiment, but it may also compromise the accuracy of the results. The addition of a few seconds to the total test time has little impact on the test subjects. In order to achieve higher sensitivity, this study made certain concessions in terms of efficiency. To obtain greater experimental efficiency, enhancements to computer programming are required. For example, adopting a dynamic test duration instead of a fixed duration, and employing a more flexible rate value rather than adhering to a fixed 50% rate are challenges that need to be addressed in subsequent steps.

A limitation of this study is that the indices established in the experiment warrant further optimization, with the aim of achieving higher detection efficiency through an increased sample size and more advanced computer-aided technology. Additionally, the age range of the subjects is restricted, necessitating further investigation to ascertain the feasibility of implementing this detection method within older or younger participants.

## Conclusion

Conventional results were as good as the eye tracking results. Stereoacuity can be evaluated utilizing a stereopsis measurement system grounded in eye tracking technology. This approach offers a novel conceptual framework for the assessment of stereoacuity in future investigations.

## Supporting information

S1 Data (PDF)
