# Supplementary material for: Comparison of stereopsis thresholds measured with conventional methods and a new eye tracking method
Source: PLoS One. 2023 Nov 2;18(11):e0293735. doi: 10.1371/journal.pone.0293735 (PMC10621823; doi:10.1371/journal.pone.0293735)
Supplement: S1 Data — (PDF) [file pone.0293735.s001.pdf]

Supplementary Material

Supplementary Table 1.

Test results of stereopsis recognition reaction time (second)

ID	Stereopsis recognition reaction time	ID	Stereopsis recognition reaction time	ID	Stereopsis recognition reaction time
1	3.119	11	3.397	21	3.503
2	2.942	12	1.076	22	3.333
3	4.796	13	2.698	23	1.803
4	4.297	14	1.421	24	3.999
5	3.919	15	2.909	25	5.366
6	3.503	16	2.874	26	5.82
7	4.152	17	1.975	27	5.302
8	4.087	18	3.086	28	5.462
9	3.095	19	3.808	29	3.656
10	2.845	20	1.821	30	4.473

Supplementary Table 2.

Test results of conventional and innovative stereopsis thresholds values (log arcsec)

ID	Conventional	Innovative	ID	Conventional	Innovative
1	1.1	1.1	31	1.4	1.2
2	1.1	1.1	32	1.5	1.8
3	1.1	1.1	33	1.5	1.5
4	1.1	1.1	34	1.5	1.7
5	1.1	1.1	35	1.5	1.4
6	1.1	1.1	36	1.7	1.4
7	1.1	1.1	37	1.7	1.1
8	1.1	1.1	38	1.7	1.7
9	1.1	1.1	39	1.7	1.2
10	1.1	1.1	40	1.7	1.8
11	1.1	1.1	41	1.7	1.8
12	1.2	1.1	42	1.7	1.1
13	1.2	1.7	43	1.7	1.1
14	1.2	1.2	44	1.7	1.4
15	1.2	1.1	45	1.7	1.2
16	1.2	1.7	46	1.7	1.5
17	1.2	1.2	47	1.7	1.7
18	1.2	1.1	48	1.7	1.2
19	1.2	1.1	49	1.7	1.2
20	1.2	1.7	50	1.4	1.8
21	1.2	1.7	51	1.4	1.8
22	1.2	1.1	52	1.4	1.2
23	1.2	1.2	53	1.4	1.4
24	1.2	1.1	54	1.4	1.7
25	1.2	1.1	55	1.4	1.2
26	1.2	1.2	56	1.5	1.7
27	1.2	1.2	57	1.5	1.8
28	1.2	1.2	58	1.5	1.7
29	1.2	1.2	59	1.5	1.7
30	1.4	1.2	60	1.8	1.8

ID	Conventio nal	Innovative	ID	Conventio nal	Innovative
61	1.8	1.8	91	1.8	1.6
62	1.7	1.5	92	1.8	1.8
63	1.7	1.7	93	1.8	1.8
64	1.7	1.7	94	1.8	1.6
65	1.7	1.5	95	1.8	2
66	1.7	1.8	96	1.2	1.1
67	1.8	1.7	97	1.2	1.2
68	1.8	1.8	98	1.5	1.3
69	1.8	1.8	99	1.5	1.3
70	1.8	1.7	100	1.7	1.3
71	1.8	2	101	1.7	1.6
72	1.8	1.8	102	1.8	2.2
73	1.5	1.4	103	1.8	2.2
74	1.5	1.3	104	1.7	1.6
75	1.5	1.7	105	1.7	1.7
76	1.5	1.5	106	1.8	1.8
77	1.5	1.3	107	2	2
78	1.5	1.5	108	2	1.7
79	1.5	1.8	109	2	1.8
80	1.5	1.3	110	2	2
81	1.6	1.7	111	2	2.2
82	1.6	1.6	112	2	2
83	1.6	1.5	113	2	1.8
84	1.6	1.4	114	2.2	2.2
85	1.6	1.8	115	2.2	2.2
86	1.6	1.4	116	2.2	2
87	1.6	1.6	117	2.2	1.8
88	1.6	1.6	118	2.2	2.2
89	1.7	1.7	119	2.2	2.2
90	1.7	1.7	120	2.2	2
